# The association between multimorbidity and food insecurity among US parents, guardians, and caregivers

**DOI:** 10.1186/s12889-025-22714-3

**Published:** 2025-04-22

**Authors:** Claire E. Branley, Melissa Goulding, Mayra Tisminetzky, Stephenie C. Lemon

**Affiliations:** 1 Department of Population and Quantitative Health Sciences, University of Massachusetts Chan Medical School, 55 Lake Ave North, 01655 Worcester, Massachusetts USA; 2 Division of Preventative and Behavioral Medicine, Department of Population and Quantitative Health Sciences, University of Massachusetts Chan Medical School, Worcester, Massachusetts USA; 3 Division of Health Systems Science, Department of Medicine, University of Massachusetts Chan Medical School, Worcester, Massachusetts USA

**Keywords:** Food insecurity, Multimorbidity, Social determinants of health, Chronic disease, Parenting

## Abstract

**Background:**

Multimorbidity among parents, guardians, and caregivers may increase the risk of household food insecurity, which would negatively impact both parents and children. However, limited research has been done to evaluate this relationship among this population. To fill this gap, we examined the association between multimorbidity and food insecurity among U.S. parents.

**Methods:**

Cross-sectional data from 2019 to 2022 of the National Health Interview Survey were analyzed. Parents, guardians, and caregivers with complete data (*N* = 26,579) were included. Multimorbidity is defined as having 2 or more conditions. In this study, multimorbidity was categorized as 2 or 3 + conditions from a sum of 11 chronic conditions: hypertension, hyperlipidemia, diabetes, arthritis, stroke, cancer, asthma, depression, anxiety, chronic obstructive pulmonary disease, and heart disease. The presence of food insecurity was defined in four nominal categories (secure, marginal food security, low food security, very low food security). Survey-weighted multinomial regression was used to assess the association of multimorbidity with food insecurity categories, controlling for sociodemographic characteristics. The association between physical versus physical-mental comorbidities and food insecurity was also analyzed.

**Results:**

The mean study sample age was 38.8 years, 51% were women and 53% were non-Hispanic White race/ethnicity. Nearly half (49%) had ≥ 1 chronic condition; 23% had 1, 14% had 2, and 13% had 3+. The most common pair of chronic conditions among parents was depression and anxiety, and most common triad was depression, anxiety, and hypertension. After controlling for potential confounders, we found that parents with 3 + conditions had a higher risk of marginal (OR 1.75, 95% CI 1.47–2.10), low (OR 2.20, 95% CI 1.75–2.75), and very low food security (OR 4.1, 95% CI 3.2–5.2) compared to parents with no conditions. Differences were seen in the odds of food insecurity among parents with mental and physical conditions, as opposed to physical comorbidities alone.

**Conclusions:**

Our findings suggest a higher risk of food insecurity in parents with multimorbidity. Parents with multimorbidity (especially those with comorbid depression and anxiety disorders) may be a key population to identify and intervene on food insecurity to improve health and well-being among US families.

**Supplementary Information:**

The online version contains supplementary material available at 10.1186/s12889-025-22714-3.

## Introduction

In 2022, approximately 40% of American adults reported multimorbidity, defined as the co-occurrence of two or more chronic conditions in an individual [1]. Persons with multimorbidity are at increased risk for longer hospital stays and premature death [2]. They also incur additional health expenses, spend more time managing appointments, and have increased physical health challenges and reduced ability to work full time [2]. This can force individuals to make difficult trade-offs on time and money spent on healthcare, utilities, housing, or food, and may lead to a state of food insecurity.

Food insecurity is defined as limited or uncertain access to adequate food and affected 12.8% of US households in 2022 [3]. Parents, guardians and caregivers, particularly single mothers, consistently have higher rates of household food insecurity compared to adult-only households [3]. Food insecurity is associated with high rates of chronic disease, healthcare underuse, and poor mental health among parents [4]. Among children, food insecurity increases risks of poor academic performance, overall poor physical and mental health, and behavioral challenges [4–8]. Therefore, preventing food insecurity is imperative to reducing long term negative health and behavioral outcomes among parents and children.

The determinants of food insecurity are complex. The rates of food insecurity among Black, Native American, and Hispanic families in the US have consistently been twice as high as White families [3]. This is due in part to systemic racism, which leads to policies and practices that decrease opportunities for education, job stability, and housing among these populations [9]. The Healthy People 2030 goals acknowledge that preventing food insecurity is an important objective to improving population health and reducing these health disparities [10]. The goals specify eliminating very low food security, which represents significant disrupted eating patterns, among households with children [10]. Unfortunately, the status for this objective is “getting worse” which indicates a clear need to identify determinants of very low food security, outside of income, education, and other demographic associations, among US households with children [10].

One potential factor that could predispose households with children to food insecurity is the health status of their caregivers. Although multimorbidity is strongly associated with aging, an estimated 1 in every 7 adults under the age of 45 have multimorbidity [2, 11]. Despite this high prevalence among younger adults, the majority of multimorbidity literature focuses on adults over 65 years old [7]. When examining the potential relationship between multimorbidity and food insecurity, past research has only focused on older adults [7, 12, 13]. There is a need for studies utilizing a family-centered approach to evaluate how multimorbidity among parents may impact the food security of the household, which would likely in turn increase the likelihood of poor health outcomes for their children. Understanding the association between multimorbidity and food insecurity can contribute to ongoing efforts among medical systems to screen, identify, and intervene on patients at high risk of experiencing food insecurity [14].

The purpose of this study was to examine the association between multimorbidity and food insecurity in a national sample of parents, guardians and caregivers of children. We utilized nationally representative data from the National Health Interview Survey to (1) describe the prevalence of multimorbidity and the most common combinations of chronic conditions among parents, guardians, and caregivers in the US and (2) evaluate the association of multimorbidity and household food insecurity among this population.

## Methods

### Data source

We conducted a cross-sectional study among a national sample of parents, guardians, and caregivers (hereafter referred to as parents). Publicly available data from the National Health Interview Survey (NHIS), which is an annual household interview survey of non-institutionalized United States residents was used. The survey monitors trends in illness and disability prevalence, severity, and impact. In households with multiple adults, only one randomly selected adult completes the survey [15]. We extracted and pooled NHIS data from the 2019–2022 survey years from the IPUMS Health Surveys website [16]. Multiple years were combined to allow for an adequate sample size of parents, and years prior to 2019 were not included due to a redesign in the NHIS questionnaire. The average response rate across these years was 54% [15]. This study received an exemption from The Committee for the Protection of Human Subjects in Research at UMass Chan Medical School.

### Study sample

The population of interest was composed of parents defined as adults 18 years and older who reported caring for at least one minor under the age of 18 years. The combined 2019–2022 NHIS survey data included 140,991 adults (18 years or older) respondents of which 28,654 reported caring for minors and therefore were included. We used a complete case analysis such that we excluded respondents if they were missing data on one or more of the following factors: age, race or ethnicity, education, current smoking status, Body Mass Index (BMI), marital status, employment status, insurance status, household food insecurity status, and complete answers to eleven questions regarding the presence of select chronic diseases (*n* = 2,075). Our final analytic sample included 26,579 parents which represented a weighted U.S. population of 70,706,411 persons.

### Multimorbidity

Multimorbidity is classically defined as 2 or more chronic conditions coexisting in the same individual [2]. NHIS consistently asked about the following 11 chronic conditions in 2019–2022 surveys: hypertension, hyperlipidemia, diabetes, arthritis, stroke, cancer, asthma, depression, anxiety, chronic obstructive pulmonary disease (COPD), and heart disease. We first classified the presence of these chronic conditions based on survey respondents answering “yes” to a question such as, “Have you ever been told by a doctor or health professional that you have hypertension?”. The presence of COPD was determined via affirmative response to ever having COPD, emphysema, or chronic bronchitis, and the presence of heart disease was determined via affirmative response to ever having a heart attack, angina pectoris, or coronary heart disease. We then tallied the number of chronic conditions present for each respondent to generate our multimorbidity exposure categories of 0, 1, 2, and 3 + chronic conditions. This categorization was used so we could examine the impact of each additional condition, although both the 2 and 3 + condition categories are considered ‘multimorbid’ [2, 7].

### Food insecurity

The primary study outcome of food insecurity was measured using a United States Department of Agricultural (USDA) 10-item screening survey for Adult Food Security [32]. This screener includes questions referencing the past 30 days, such as “We worried whether food would run out before we could get money to buy more” [3]. NHIS counts affirmative responses and categorizes households into food secure (0 positive responses), marginal food security (1–2 responses), low food security (3–5 responses), and very low food security (6–10 responses) categories. We maintained these categories as the outcome for this study. Although the NHIS does not measure food insecurity at the child level, there is a strong correlation between poor health outcomes and food insecurity at the household level, even if the child is not reported to be reducing food intake or skipping meals [17].

### Covariates

We selected potentially confounding factors a priori based on prior literature [2, 3, 7, 18]. A directed acyclic graph visually depicting these relationships is available in Supplemental Fig. 1. We included demographic variables that are associated with both food insecurity and multimorbidity, namely gender, age, and marital status (defined as married, widowed/divorced/separated, living with a partner, or never married/single). Self-reported race/ethnicity (defined by the NHIS as Non-Hispanic White, NH Black/African American, NH American Indian/Alaska Native, NH Asian, Other race/multiple race, Hispanic/Mexican or Hispanic/other) was included as a covariate as a proxy for racism [9]. Socioeconomic status, which includes educational attainment (defined as less than high school, high school/GED, some college, or bachelor’s degree/higher), income (ratio of family income to poverty threshold), and employment status (current full-time employment, yes/no) were used. Insurance status was also included, defined as private, Medicare, public, or no insurance. A sensitivity analysis was done to evaluate the role of lack of recent healthcare utilization by excluding those who reported they had not seen a doctor in the last year and comparing results to the original population.

We also included variables that are related to common chronic conditions in our multimorbidity definition and are also associated with food insecurity. These included history of cigarette smoking (operationalized as never smoker, former smoker, and current everyday/someday smoker) and Body Mass Index (operationalized into BMI > 30 indicating obesity, BMI between 25 and 30 denoting overweight, and BMI < 25 denoting normal or underweight).

### Statistical analysis

All analyses used NHIS sampling weights and stratum indicators to account for the complex survey design, including accounting for a high increase in non-response rates in 2020. We first examined descriptive characteristics of the study sample. Continuous data were summarized as means with standard deviations, and bivariate or categorical data were summarized as percentages. Demographic characteristics were compared across the multimorbidity exposure categories using chi square tests. The overall prevalence of chronic conditions in the entire study sample, as well as the prevalence of more common condition combinations among the multimorbid groups, were described. For our regression analysis we first explored ordinal regression, however the model was rejected as the proportional odds assumption was violated and a multinomial regression was selected instead. Using survey weighted multinomial regression we calculated unadjusted and adjusted odds of food insecurity. In a secondary analysis, we explored the role of physical-mental comorbidity by further stratifying the exposure groups into parents with only depression, only anxiety, depression or anxiety and one physical condition, depression or anxiety and 2 + physical conditions, and parents with 1, 2, and 3 + physical conditions (no depression or anxiety). The adjusted multivariable models for both analyses included sociodemographic factors selected a priori as described above [17]. Covariates included in the multivariable models were age, sex, race/ethnicity, marital status, education, income, insurance status, employment status, smoking history, and BMI. Results are expressed as odds ratios with accompanying 95% confidence intervals. All analyses were carried out using STATA 17.0 software.

## Results

### Study population characteristics

The average age of the study sample was 38.8 years, approximately one-half (51%) were women and non-Hispanic White (53%). Approximately one-third of the sample had obtained a bachelor’s degree or higher, 64% were married, and the majority (76%) were employed. Approximately half (51%) had no chronic conditions, while 23% had one, 14% had two and 12% had three or more.

Parents with multimorbidity had a higher average age (43.7 years in the 3 + condition group compared to 35.8 in no-condition group), and a higher proportion female, non-Hispanic White, not currently employed, and widowed, divorced, or separated compared to parents with no chronic conditions (Table 1). Parents with multimorbidity reported higher rates of living with incomes under 200% of the federal poverty level, as well as being enrolled in Medicare or Medicaid; however, parents with 0 conditions were more likely to have no insurance than those in the 2 or 3 + condition group. Parents with multimorbidity were also less likely to have a bachelor’s degree or higher and were more likely to be a current smoker and have a BMI > 30.

**Table 1:** Descriptive statistics of parents, guardians, and caregivers by number of chronic conditions from National health interview survey years 2019–2022 (*n* = 26,579).a

	Chronic Conditions			
	0 conditions	1 condition	2 conditions	3 + conditions
Sample size	13,222 (50%)	6,235 (23%)	3,694 (14%)	3,428 (13%)
Weighted population	36,025,681	16,316,208	9,587,068	4,780,518
Age years (mean, SD)	35.8 (9.3)	40.3 (10.4)	41.4 (11.4)	43.7 (12.4)
Female (weighted %)	52.1	52.3	57.8	61.9
Race/Ethnicity (weighted %)				
Non-Hispanic White	48.6	55.4	60.9	61.5
NH Black or African American	11.8	12.3	10.5	12.1
NH American Indian/Alaska Native	1.3	1.3	1.3	2.9
NH Asian	8.7	6.6	5.1	3.6
Other and/or multiracial	1.5	1.6	1.9	1.4
Hispanic, Mexican	18.3	15.1	12.4	11.6
Hispanic, other	9.7	7.7	8.0	7.0
Education (weighted %)				
Less than high school	12.5	11.2	12.3	15.9
High school diploma/GED	27.1	23.6	24.5	27.5
Some college	27.6	28.1	30.9	32.8
Bachelor’s degree or higher	32.7	37.2	32.3	23.8
Work Status: Employed (weighted %)	79.0	79.2	74.3	56.7
Federal income to poverty level (weighted %)				
> 400%	34.1	38.4	35.5	24.4
200–400%	31.6	31.5	30.1	29.4
< 200%	34.3	30.1	34.4	46.2
Insurance status				
Private	63.5	66.0	61.2	43.5
Medicare	0.8	2.3	4.3	11.4
Public	18.3	19.4	24.1	36.0
No insurance	17.3	12.3	10.5	9.2
Marital status (weighted %)				
Married	63.4	67.8	64.6	58.8
Living with partner	10.2	9.5	8.6	9.5
Widowed/divorced/separated	5.1	8.5	10.9	18.9
Never married	21.3	14.3	15.9	12.9
Smoking status (weighted %)				
Never smoker	76.6	70.7	62.7	52.1
Former smoker	14.2	18.5	22.8	27.4
Current everyday/someday smoker	9.2	10.9	14.5	20.6
BMI Category (weighted %)^b^				
Underweight/Normal weight ( < = 24.9)	38.4	28.2	25.0	17.5
Overweight (25-29.9)	35.0	35.2	31.3	29.5
Obese (30+)	26.6	36.6	43.7	53

^a^ Percentages may not sum to 100% due to rounding.

^b^ Calculated from self-reported height and weight.

### Burden of specific conditions and multimorbidity

The absolute prevalences of the most common chronic disease among parents in this sample are available in Table 2. Among parents with 2 conditions (*N* = 3,701) the most frequent combination of conditions was depression and anxiety (29%) (Table 3). Among those with three conditions (*N* = 1,879), 24% had depression, anxiety, and hypertension.

**Table 2:** Prevalence of chronic diseases among parents, guardians, and caregivers in the study sample. Data collected via self-report from the National health interview survey, years 2019–2022, (*n* = 26,579)

Condition	Prevalence (weighted %)
Hypertension	19.3
High cholesterol	15.5
Depression	15.3
Anxiety	15.2
Arthritis	10.2
Asthma	7.6
Diabetes	5.2
Cancer	3.8
Heart disease (angina, MI^a^, or CAD^b^)	2.2
COPD ^c^	2.1
Stroke	1.2

^a^ Myocardial infarction

^b^ Coronary artery disease

^c^ Chronic obstructive pulmonary disease

**Table 3:** Prevalence of the top three most frequent chronic conditions or combinations of conditions among parents, guardians, and caregivers in the study sample with one, two, or three chronic conditions. Data collected via self-report from the National health interview survey, years 2019–2022

Parents with one chronic condition (*n* = 6,235)	Prevalence (weighted %)
Hypertension	26
High cholesterol	20
Asthma	12
Parents with two conditions (*n* = 3,694)	
Depression and anxiety	30
Hypertension and high cholesterol	14
Hypertension and diabetes	4
Parents with three conditions (*n* = 3,428)	
Depression, anxiety, and hypertension	24
Depression, anxiety, and arthritis	20
Depression, anxiety, and asthma	14

### Multimorbidity and food insecurity

The frequency of food insecurity (marginal, low, or very low food security) increased with an increasing number of self-reported chronic conditions: 6% among those who did not have any of the 11 conditions reported food insecurity, compared with 8% among those with one condition, 11% among those with 2 chronic conditions and 17% among those with 3 + chronic conditions. The results of the adjusted multinomial logistic regression model of the association between multimorbidity and food insecurity among parents are shown in Table 4. If a parent reported having one condition compared to no conditions, the adjusted odds (aOR) of marginal food security was 1.26 times higher (95% CI 1.09–1.46) than high food security. However, for parents that reported only two conditions compared to those with no conditions, the aOR of marginal food security was 1.10 times higher than high food security, although this was not statistically significant (95% CI 0.90, 1.34). The aOR of marginal food security were highest among the 3 + condition group (aOR 1.75, 95% CI 1.47, 2.10) compared to the 0-condition group. The odds of low food security was similar for those who have 1 condition (aOR 1.70, 95% CI 1.42, 2.04) and 2 conditions (aOR 1.70, 95% CI 1.38, 2.09) as compared to parents with 0 conditions, but were 2.20 times higher (95% CI 1.75, 2.75) in the 3+-condition group. A similar pattern emerged when predicting very low food security among households. Parents with 3 + conditions had 4.05 (95% CI 3.16–5.18) times higher odds of reporting very low food security compared to those with 0 conditions.

**Table 4:** Weighted multinomial logistic regression models of the association between chronic conditions and household food insecurity among parents, guardians, and caregivers. Data obtained via self-report from the National health interview survey, years 2019–2022, *N* = 26,579.a

	Marginal food security		Low food security		Very low food security	
	Adjusted Odds Ratio [95% CI]	P-value	Adjusted Odds Ratio [95% CI]	P-value	Adjusted Odds Ratio [95% CI]	P-value
Number of chronic conditions						
0	Ref.		Ref.		Ref.	
1	1.26 [1.09, 1.46]	0.002	1.70 [1.42,2.04]	0.000	1.70 [1.33,2.18]	0.000
2	1.10 [0.90,1.34]	0.325	1.70 [1.38,2.09]	0.000	2.08 [1.58, 2.73]	0.000
3+	1.75 [1.47, 2.10]	0.000	2.20 [1.75, 2.75]	0.000	4.05 [3.16, 5.18]	0.000

^a^ Adjusted for race/ethnicity, age, gender, educational attainment, ratio of income to federal poverty level, insurance status, marital status, BMI, smoking history, and employment

Given the high proportion of parents that reported either depression, anxiety, or both, further analyses delineating the association of different combinations of conditions was done. The results of this multivariable regression are available in Table 5. First, we compared the relative effects of having depression alone, anxiety alone, or one physical condition compared to having no conditions. This revealed that parents with depression alone had significantly higher odds of marginal (aOR 2.01, 95% CI 1.41, 2.85), low (aOR 2.46, 95% CI 1.59, 3.82), and very low (aOR 2.15, 95% CI 1.27, 3.65) food security compared to parents with no conditions. Those with either two physical conditions, or two conditions with a mental-physical comorbidity, were not significantly associated with marginal food insecurity but did report significantly higher odds of low and very low food security. Those with depression or anxiety and two other conditions had significantly increased risk of marginal (aOR 1.82, 95% CI 1.48, 2.23), low (aOR 2.56, 95% CI 2.01, 3.26), and very low (aOR 4.57, 95% CI 3.55, 5.88) food security.

**Table 5:** Weighted multinomial logistic regression models of the association between different types of morbidity combinations (mental, physical, or mental-physical) and household food insecurity among parents, guardians, and caregivers. Data obtained via self-report from the National health interview survey, years 2019–2022, *N* = 26,579.a

	Marginal food security		Low food security		Very low food security	
	Adjusted Odds Ratio [95% CI]	P-value	Adjusted Odds Ratio [95% CI]	P-value	Adjusted Odds Ratio [95% CI]	P-value
No conditions (*N* = 13,222)	Ref.		Ref.		Ref.	
Depression alone (*N* = 642)	2.01 [1.41, 2.85]	0.000	2.46 [1.59, 3.82]	0.000	2.15 [1.27, 3.65]	0.005
Anxiety alone (*N* = 701)	1.48 [1.05, 2.09]	0.024	1.37 [0.84, 2.25]	0.208	0.68 [0.37, 1.25]	0.213
One physical condition (*N* = 4,892)	1.14 [0.97, 1.34]	0.121	1.63 [1.34, 1.98]	0.000	1.77 [1.02, 2.63]	0.000
Depression or anxiety and one other condition, two conditions total (*N* = 1,988)	1.11 [0.87, 1.42]	0.384	1.85 [1.44, 2.78]	0.000	2.38 [1.76, 3.22]	0.000
Two physical conditions (*N* = 1,706)	1.08 [0.83, 1.41]	0.556	1.51 [1.12, 2.04]	0.007	1.63 [1.02, 2.63]	0.043
Depression or anxiety and two other conditions, 3 + conditions total (*N* = 2,454)	1.82 [1.48, 2.23]	0.000	2.56 [2.01, 3.26]	0.000	4.57 [3.55, 5.88]	0.000
Three physical conditions (*N* = 974)	1.59 [1.20, 2.10]	0.001	1.42 [0.99, 2.02]	0.052	2.69 [1.74, 4.16]	0.000

^a^ Adjusted for race/ethnicity, age, gender, educational attainment, ratio of income to federal poverty level, insurance status, marital status, BMI, smoking history, and employment

### Sensitivity analysis

Among the parents that reported having no insurance, approximately 50% had also not seen a physician in the last year. We conducted a sensitivity analysis that excluded those that reported they had not seen a physician in the last year (*n* = 4,637). We found that excluding this population did not appreciably change the aORs (Supplemental Table 1).

## Discussion

This study is among the first to assess the association between multimorbidity and food insecurity among parents in the US. The results of this nationally representative study suggest that parents, inclusive of guardians and caregivers, living with 3 or more chronic conditions had higher odds of marginal, low, and very low food security compared to parents with no conditions. Furthermore, results suggest the strength of this association increases with a dose-response relationship, with increasing number of chronic conditions being associated with greater odds of food insecurity. Our results indicate that in efforts to eliminate very low food security among children, identifying parents with multiple chronic conditions could be a key strategy.

Our results are consistent with a previous meta-analysis of five studies which found that multimorbidity was associated with 2.58 times higher odds of food insecurity [7]. Four of the five studies included in this meta-analysis restricted their population to adults > 50 years old, and there was wide variation in the definition of multimorbidity, with some comparing 2 vs. 0–1 chronic conditions, while others defined multimorbidity as 5 or more conditions [7]. Our study adds to these results by showing that despite being an overall younger population, parents with multiple common chronic conditions face higher odds of food insecurity. Additionally, several of the prior studies in older adults used a two-category definition of food insecurity. By utilizing four categories, our results provide important nuance to the experience of food insecurity. Our findings indicate that parents with 3 + conditions had 4 times the odds of experiencing very low food security, and that parents with comorbid physical and mental health conditions were associated with the highest odds of very low food security. Very low food security signifies disrupted eating patterns and reduced food intake for at least one person in the household [3]. Although parents often shield their children from the impacts of food security by altering diets or cutting their own portions, a household with very low food security is most likely to show severe negative health implications for children as well as adults; therefore, it is important to highlight these populations which may be at highest risk [19].

There are multiple potential mechanisms through which the relationship between multimorbidity and food insecurity could be mediated, such as the increased time and money required to manage multimorbid conditions. Prior evidence has shown that living in a food insecure household predisposes adults to several chronic conditions which can lead to multimorbidity, particularly among older adults [4, 5]. We believe the relationship between multimorbidity and food insecurity is likely to be bidirectional. A previously described model of “cumulative complexity” for patients with multimorbidity applies to this study [20]. For example, a parent with hypertension, anxiety, and depression may be taking multiple medications, managing appointments for more than one provider, and attempting to implement lifestyle recommendations for multiple conditions. It has been shown that the combination of conditions leads to an exponential increase in healthcare expenditures in older adults, as well as increased odds of a catastrophic healthcare expense that poses a high economic risk for households [2, 21]. The cumulative burden of managing these conditions can lead to food insecurity as parents may be forced to decide how to spend their limited time and resources: on medicine, healthcare, or food [22, 23].

This burden may also explain the differences seen in parents with physical comorbidities versus physical-mental comorbidities. In our study depression and anxiety were nearly as prevalent as high cholesterol and hypertension. Furthermore, depression and anxiety were the most frequent combinations of disease in the 2 and 3-condition groups. A parent with depression alone had higher odds of food insecurity than parents with multiple physical conditions. Our findings expand on a previous paper which found that reporting one chronic disease was not associated with food insecurity, but adults who had chronic disease with depression had significantly increased odds of food security [34]. In contrast, we found that even one physical chronic condition is associated with higher odds of food insecurity among parents, and that these odds increase with additional physical conditions and the combination of depression and anxiety.

It is possible that this association occurs because depression and anxiety are a risk factor for comorbidity in general. Previous studies that have linked pre-existing depression and anxiety to the accumulation of chronic conditions, such as diabetes and hypertension [24, 25]. Additionally, co-occurring depression and anxiety have been more frequently identified in younger populations with multimorbidity compared to older ones [26]. This mental-physical comorbidity may be particularly important to consider for parents, as qualitative data describes the important connection between the toxic stress of poverty among parents as they juggle their own health needs with those of their children, and how this contributes to chronic depressive symptoms [27]. Future qualitative data specifically from the population of parents experiencing physical and mental comorbidity is needed, both to identify mediating factors between comorbidities and food insecurity as well as to aid efforts in screening for food insecurity and connecting parents to resources that can intervene.

Although our study does not include child-level outcomes, we must consider that multimorbidity, particularly physical-mental comorbidity, and food insecurity in parents may also have important implications for the child they care for. Over extended periods of time, exposure to food insecurity has important negative impacts on children’s social, emotional, and behavioral wellbeing [4, 5, 19, 28, 33]. Multimorbidity in the parents can translate to a cycle of ill health in the family, as children living in food insecure households are more also likely to have a chronic health condition, and experience delayed medical and dental care due to cost [19]. Having chronically ill parents may also be associated with worse behavioral or academic outcomes irrespective of food insecurity [29]. Multiple studies have also demonstrated how parents attempt to shield their children from the effects of food insecurity, including skipping portions, decreasing diet quality, or seeking assistance from emergency food resources such as food pantries [27, 30]. Therefore, while parents may use coping mechanisms to shield their children from the reality of food insecurity, chronically ill parents may be less able to do so. The combined effect of co-occurring multimorbidity and food insecurity may also further exacerbate health disparities among children and youth by race, ethnicity, and socioeconomic status [31].

Strengths of this study include our use of a robust, contemporary, and nationally representative dataset to analyze the relationship between multimorbidity and food insecurity. The population of parents in this study has novelty after previous studies focused mostly on older adults [7]. The inclusion of mental health conditions in our operational definition of multimorbidity also demonstrated the importance of considering holistic health measures when predicting food insecurity. Limitations in this study include the cross-sectional design, which limits the ability to draw conclusions about the temporal nature of the relationship between multimorbidity and food insecurity. Additionally, NHIS data are limited to self-reported conditions, which means that we were unable to distinguish between active diagnoses, disease remittance, and undiagnosed conditions, or severity of disease.

## Conclusions

The present study suggests a strong association between the number of chronic conditions experienced by parents and household food insecurity status, which has negative implications for both parents and children. Efforts are underway across U.S. public health and medical systems to both effectively address multimorbidity by increasing coordination of services, as well as to reduce food insecurity [14]. Our results highlight that parents with multimorbidity may be a crucial population for these efforts to target, especially as we work towards the goal of eliminating very low food security among children. Future research should continue to elucidate the relationships between multimorbidity and food insecurity specifically among those with mental health conditions, ideally examining the severity of mental health symptoms and effect modification of current or former mental health treatment. Increased awareness and connection of parents to programs that address food insecurity, as well as increased access to mental health support services, may positively impact the parent by reducing the burden of multimorbidity and confer positive benefits to their children.

## Electronic supplementary material

Below is the link to the electronic supplementary material.

Supplementary Material 1

Supplementary Material 2

## Data Availability

The datasets analyzed in the current study are free and available on the IPUMS Health Surveys data repository, accessible at https://healthsurveys.ipums.org/.

## Abbreviations

NHIS: National Health Interview Survey
BMI: Body Mass Index
COPD: Chronic Obstructive Pulmonary Disease
US: United States
USDA: United States Department of Agriculture
OR: Odds Ratio

## References

[1] CDC’s National Center for Chronic Disease Prevention and Health Promotion (NCCDPHP). Chronic Diseases in America [Internet]. Available from: https://www.cdc.gov/chronicdisease/pdf/infographics/chronic-disease-in-america-H.pdf

[2] Skou ST, Mair FS, Fortin M, Guthrie B, Nunes BP, Miranda JJ, et al. Multimorbidity Nat Rev Dis Primer. 2022;8(1):48. 10.1038/s41572-022-00376-4 PMC7613517 35835758

[3] Rabbitt MP. Household Food Security in the United States in 2022.

[4] Gundersen C, Ziliak JP. Food insecurity and health outcomes. Health Aff Proj Hope. 2015;34(11):1830–9. 10.1377/hlthaff.2015.0645 26526240

[5] Cain KS, Meyer SC, Cummer E, Patel KK, Casacchia NJ, Montez K, et al. Association of food insecurity with mental health outcomes in parents and children: A systematic review. Acad Pediatr. 2022;22(7):1105–14. 35577282 10.1016/j.acap.2022.04.010 PMC10153634

[6] Shankar P, Chung R, Frank DA. Association of food insecurity with children’s behavioral, emotional, and academic outcomes: A systematic review. J Dev Behav Pediatr. 2017;38(2):135. 28134627 10.1097/DBP.0000000000000383

[7] Kantilafti M, Giannakou K, Chrysostomou S. Multimorbidity and food insecurity in adults: A systematic review and meta-analysis. PLoS ONE. 2023;18(7):e0288063. 37410753 10.1371/journal.pone.0288063 PMC10325088

[8] Gucciardi E, Vahabi M, Norris N, Del Monte JP, Farnum C. The intersection between food insecurity and diabetes: A review. Curr Nutr Rep. 2014;3(4):324–32. 25383254 10.1007/s13668-014-0104-4 PMC4218969

[9] Odoms-Young A, Bruce MA. Examining the impact of structural racism on food insecurity: implications for addressing Racial/Ethnic disparities. Fam Community Health. 2018;41:S3. 10.1097/FCH.0000000000000183. 29461310 10.1097/FCH.0000000000000183 PMC5823283

[10] US Department of Health and Human Services. Healthy People 2030 - Food Insecurity [Internet]. [cited 2024 Jul 1]. Available from: https://health.gov/healthypeople/priority-areas/social-determinants-health/literature-summaries/food-insecurity

[11] Barnett K, Mercer SW, Norbury M, Watt G, Wyke S, Guthrie B. Epidemiology of Multimorbidity and implications for health care, research, and medical education: a cross-sectional study. Lancet. 2012;380(9836):37–43. 22579043 10.1016/S0140-6736(12)60240-2

[12] Gregory CA. Food Insecurity, Chronic Disease, and Health Among Working-Age Adults.

[13] Ho ISS, Azcoaga-Lorenzo A, Akbari A, Black C, Davies J, Hodgins P, et al. Examining variation in the measurement of Multimorbidity in research: a systematic review of 566 studies. Lancet Public Health. 2021;6(8):e587–97. 34166630 10.1016/S2468-2667(21)00107-9

[14] Renaud J, McClellan SR, DePriest K, Witgert K, O’Connor S, Abowd Johnson K, et al. Addressing health-Related social needs via community resources: lessons from accountable health communities: study examines how the accountable health communities model addresses health-related social needs via community resources. Health Aff (Millwood). 2023;42(6):832–40. 37196207 10.1377/hlthaff.2022.01507

[15] National Center for Health Statistics. CDC. National Health Interview Survey (NHIS) [Internet]. Available from: https://www.cdc.gov/nchs/hus/sources-definitions/nhis.htm

[16] Lynn A, Blewett, Julia A, Rivera Drew ML, King, Kari CW, Williams A, Chen S, Richards et al. IPUMS Health Surveys: National Health Interview Survey, Version 7.3. 2023.

[17] Cook JT, Black M, Chilton M, et al. Are food insecurity’s health impacts underestimated in the U.S. Population?? Marginal food security also predicts adverse health outcomes in young U.S. Children and mothers. Adv Nutr. 2013;4(1):51–61. 10.3945/an.112.003228. 23319123 10.3945/an.112.003228 PMC3648739

[18] Alshakhs M, Jackson B, Ikponmwosa D, Reynolds R, Madlock-Brown C. Multimorbidity patterns across race/ethnicity as stratified by age and obesity. Sci Rep. 2022;12(1):9716. 35690677 10.1038/s41598-022-13733-w PMC9188579

[19] Thomas MMC, Miller DP, Morrissey TW. Food insecurity and child health. Pediatrics. 2019;144(4):e20190397. 31501236 10.1542/peds.2019-0397

[20] Shippee ND, Shah ND, May CR, Mair FS, Montori VM. Cumulative complexity: a functional, patient-centered model of patient complexity can improve research and practice. J Clin Epidemiol. 2012;65(10):1041–51. 22910536 10.1016/j.jclinepi.2012.05.005

[21] Lehnert T, Heider D, Leicht H, Heinrich S, Corrieri S, Luppa M, et al. Review: health care utilization and costs of elderly persons with multiple chronic conditions. Med Care Res Rev MCRR. 2011;68(4):387–420. 21813576 10.1177/1077558711399580

[22] Berkowitz SA, Seligman HK, Choudhry NK. Treat or eat: food insecurity, Cost-related medication underuse, and unmet needs. Am J Med. 2014;127(4):303–e3103. 24440543 10.1016/j.amjmed.2014.01.002

[23] Shapira S, Teschner N. No heat, no eat: (Dis)entangling insecurities and their implications for health and well-being. Soc Sci Med. 2023;336:116252. 37769511 10.1016/j.socscimed.2023.116252

[24] Bobo WV, Grossardt BR, Virani S, St Sauver JL, Boyd CM, Rocca WA. Association of depression and anxiety with the accumulation of chronic conditions. JAMA Netw Open. 2022;5(5):e229817. 35499825 10.1001/jamanetworkopen.2022.9817 PMC9062691

[25] Kaur H, Scholl JC, Owens-Gary M. Depression and Diabetes in Workers Across the Life Span: Addressing the Health of America’s Workforce— Behavioral Risk Factor Surveillance System, 2014–2018. 2022;35(2). 10.2337/ds21-0022 PMC9160556 35668882

[26] Álvarez-Gálvez J, Ortega-Martín E, Carretero-Bravo J, Pérez-Muñoz C, Suárez-Lledó V, Ramos-Fiol B. Social determinants of Multimorbidity patterns: A systematic review. Front Public Health. 2023;11:1081518. 37050950 10.3389/fpubh.2023.1081518 PMC10084932

[27] Knowles M, Rabinowich J, Ettinger de Cuba S, Cutts DB, Chilton M. Do you wanna breathe or eat?? Parent perspectives on child health consequences of food insecurity, Trade-Offs, and toxic stress. Matern Child Health J. 2016;20(1):25–32. 26156827 10.1007/s10995-015-1797-8 PMC4712223

[28] Jyoti DF, Frongillo EA, Jones SJ. Food insecurity affects school children’s academic performance, weight gain, and social skills. J Nutr. 2005;135(12):2831–9. 16317128 10.1093/jn/135.12.2831

[29] Sieh DS, Visser-Meily JMA, Meijer AM. Differential outcomes of adolescents with chronically ill and healthy parents. J Child Fam Stud. 2013;22(2):209. 23335841 10.1007/s10826-012-9570-8 PMC3548090

[30] Taylor EA, Foster JS, Mobley AR. Examining factors related to the food Insecurity–Obesity paradox in Low-Income mothers and fathers. Food Nutr Bull. 2021;42(2):309–16. 34002624 10.1177/03795721211011133

[31] Hoffmann JA, Alegría M, Alvarez K, Anosike A, Shah PP, Simon KM, et al. Disparities Pediatr Mental Behav Health Conditions Pediatr. 2022;150(4):e2022058227. 10.1542/peds.2022-058227 PMC9800023 36106466

[32] Coleman-Jensen A, Nord M, September. U.S. Adult Food Security Survey Module. 2012. https://www.ers.usda.gov/sites/default/files/_laserfiche/DataFiles/50764/26623_ad2012.pdf?v=3672.4

[33] Pourmotabbed A, Moradi S, Babaei A, et al. Food insecurity and mental health: a systematic review and meta-analysis. Public Health Nutr. 2020;23(10):1778–90. 10.1017/S136898001900435X. 32174292 10.1017/S136898001900435X PMC10200655

[34] O’Neal LJ, Jo A, Scarton L, Bruce MA. Food insecurity is associated with Mental–Physical comorbidities among U.S. Adults: NHANES 2013 to 2016. Int J Environ Res Public Health. 2022;19(3):1672. 10.3390/ijerph19031672. 35162697 10.3390/ijerph19031672 PMC8835150

