# Supplementary material for: The association between multimorbidity and food insecurity among US parents, guardians, and caregivers
Source: BMC Public Health. 2025 Apr 22;25:1487. doi: 10.1186/s12889-025-22714-3 (PMC12013002; doi:10.1186/s12889-025-22714-3)
Supplement: Supplementary file 1 — Supplementary Material 1 [file 12889_2025_22714_MOESM1_ESM.docx]

Supplementary Table 1: Results from a sensitivity analysis that excluded individuals that did not report seeing a doctor within the last 12 months (n=4,637). The two tables below show the original results and the results among parents who have seen a physician, adjusting for the same variables. We conclude that there is no appreciable difference in results when excluding these parents, and have therefore retained them in the analysis.

| Original results N=26,579.^a^ | | | |
| --- | --- | --- | --- |
|  | Marginal food security | Low food security | Very low food security |
|  | Adjusted Odds Ratio [95% CI] | Adjusted Odds Ratio [95% CI] | Adjusted Odds Ratio [95% CI] |
| Number of chronic conditions |  |  |  |
| 0 | Ref. | Ref. | Ref. |
| 1 | 1.26 [1.09, 1.46]** | 1.70 [1.42,2.04]** | 1.70 [1.33,2.18]** |
| 2 | 1.10 [0.90,1.34] | 1.70 [1.38,2.09]** | 2.08 [1.58, 2.73]** |
| 3+ | 1.75 [1.47, 2.10]** | 2.20 [1.75, 2.75]** | 4.05 [3.16, 5.18]** |
| ^a^ Adjusted for race/ethnicity, age, gender, educational attainment, ratio of income to federal poverty level, insurance status, marital status, BMI, smoking history, and employment  **p-value <0.001 | | | |

| New results excluding those that have not seen a physician in the last year N=21,570.^a^ | | | |
| --- | --- | --- | --- |
|  | Marginal food security | Low food security | Very low food security |
|  | Adjusted Odds Ratio [95% CI] | Adjusted Odds Ratio [95% CI] | Adjusted Odds Ratio [95% CI] |
| Number of chronic conditions |  |  |  |
| 0 | Ref. | Ref. | Ref. |
| 1 | 1.27 [1.08, 1.50]* | 1.54 [1.24,1.90]** | 1.56 [1.17,2.09]* |
| 2 | 1.13 [0.91,1.40] | 1.76 [1.40,2.21]** | 1.90 [1.40, 2.58]** |
| 3+ | 1.76 [1.46, 2.13]** | 2.27 [1.80, 2.88]** | 3.90 [2.95, 5.15]** |
| ^a^ Adjusted for race/ethnicity, age, gender, educational attainment, ratio of income to federal poverty level, insurance status, marital status, BMI, smoking history, and employment  **p-value <0.001 | | | |
