# Supplementary figures and images for: The association between multimorbidity and food insecurity among US parents, guardians, and caregivers
Source: BMC Public Health. 2025 Apr 22;25:1487. doi: 10.1186/s12889-025-22714-3 (PMC12013002; doi:10.1186/s12889-025-22714-3)

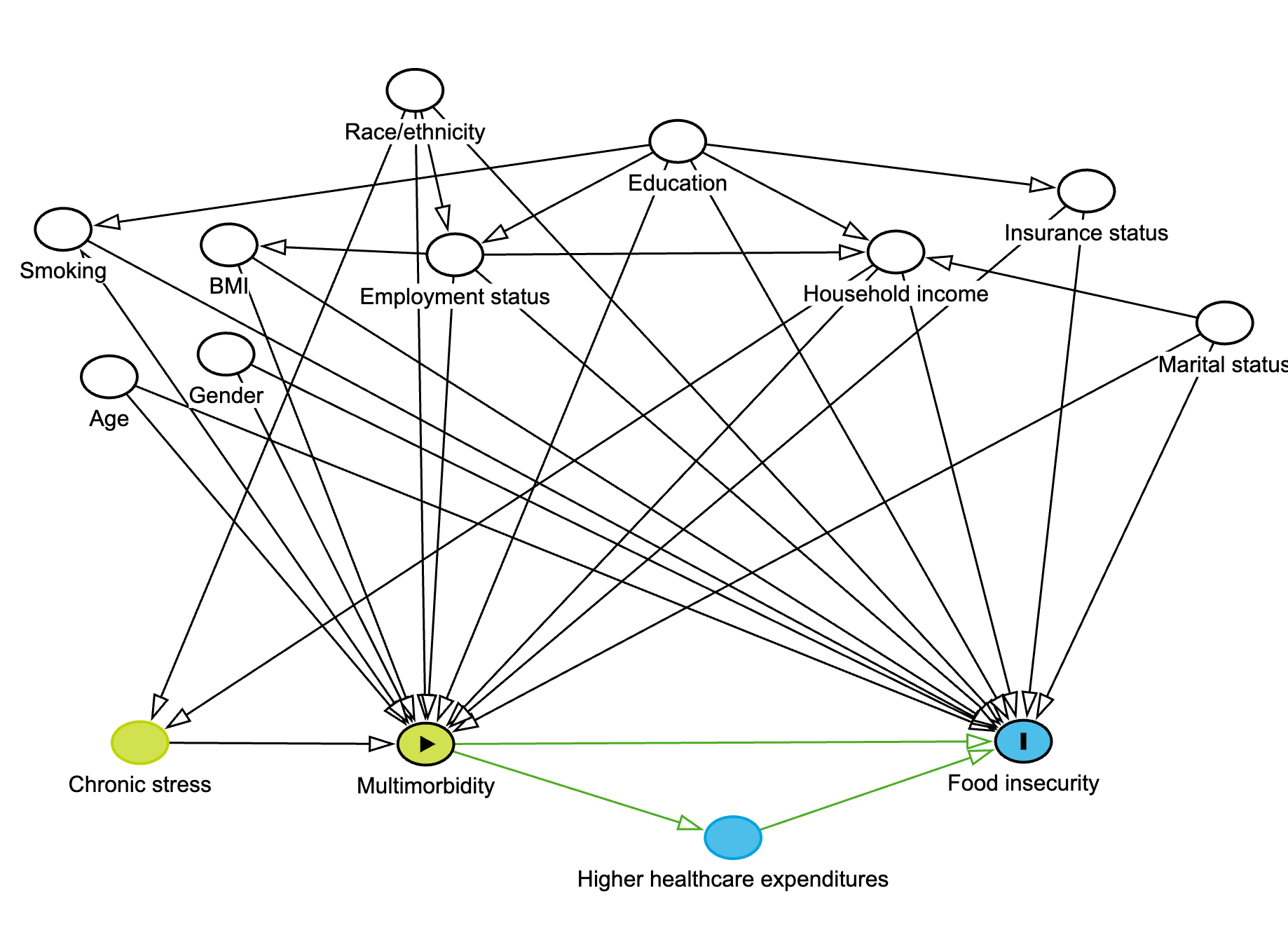

Supplement: Supplementary file 2 — Supplementary Material 2 [file 12889_2025_22714_MOESM2_ESM.png]
